# Editorial: Multi-omics and computational biology in horticultural plants: From genotype to phenotype

**DOI:** 10.3389/fpls.2022.1073266

**Published:** 2022-11-16

**Authors:** Suvendu Mondal, Hui Song, Liangsheng Zhang, Yunpeng Cao

**Affiliations:** ^1^ Nuclear Agriculture and Biotechnology Division, Bhabha Atomic Research Centre, Mumbai, India; ^2^ Homi Bhabha National Institute, Training School Complex, Anushaktinagar, Mumbai, India; ^3^ Key Laboratory of National Forestry and Grassland Administration on Grassland Resources and Ecology in the Yellow River Delta, College of Grassland Science, Qingdao Agricultural University, Qingdao, China; ^4^ Genomics and Genetic Engineering Laboratory of Ornamental Plants, College of Agriculture and Biotechnology, Zhejiang University, Hangzhou, China; ^5^ CAS Key Laboratory of Plant Germplasm Enhancement and Specialty Agriculture, Wuhan Botanical Garden, Chinese Academy of Sciences, Wuhan, China

**Keywords:** genomics, Transcriptome, genome sequencing, metabolomics, marker-trait association

Horticultural plants complement our food requirements from major agronomic crops by providing a vast range of bioactive compounds, vitamins, and minerals along with carbohydrates, reducing sugars, organic acids, proteins, and fats. They play an important role for humans by providing herbal medicines, beverages, vegetables, fruits, spices, and ornamentals. In recent years many horticultural plant genomes have been sequenced (Chen et al., 2019; Zhang et al., 2020; Liang et al., 2022) and multi-omics technologies have surfaced marker-trait association, gene expression patterns, differential gene and protein abundance along with the understanding of regulatory RNA (Hermanns et al., 2020; Luo et al., 2021). In a nutshell, high-throughput technologies have revolutionized the time scale and power of detecting insights into physiological changes and biological mechanisms in plants (Zhang and Hao, 2020; Li et al., 2022; Liang et al., 2022). All sequencing data and tools have helped us better understand the evolutionary histories of plants and provide genotype resources for molecular studies on economically important traits (Zhang et al., 2020; Zhou G. et al., 2022). The integration of these -omics technologies (e.g., genomics, transcriptomics, proteomics, metabolomics, lipidomics, ionomics, and redoxomics, etc.) is currently at the forefront of plant research. The genomes of horticultural plants are highly diverse and complex, often with a high degree of heterozygosity and polyploidy, such as *Solanum tuberosum*, modern roses, *Chrysanthemum*, and *Eustoma grandiflorum* (Hibrand Saint-Oyant et al., 2018; Liang et al., 2022; Sun et al., 2022; Wen et al., 2022). Novel computational methods need to be developed to take advantage of state-of-the-art genomic technologies. The mining of multi-omics data and the development of new computational biology approaches will help to generate reliable data and efficient analysis of plant traits. This, in turn, will aid to manipulate crop yield to address the ever-growing demand for quality food. The integration of multi-omics data and computational biology technologies constructs biological networks to identify traits that can be further applied toward horticultural crop breeding and generated more productive crop varieties. The present Research Topic on “*multi-omics and computational biology in horticultural plants: from genotype to phenotype*” aims to combine high-throughput omics and computational biology technologies to find a coherently matching genotype-to-phenotype relationship or marker-trait association in horticultural crops.

This Research Topic has published 31 articles. Of them, three are reviews and the remaining 28 are research papers. Of these, three reported genome sequencing of horticultural crops, two contain research on fruit crops, three are on vegetables, two are on spices, six are on ornamental crops, three on other industrial crops, and the remaining twelce are on model crop species.

## Genome sequencing of horticultural plants

A genome is the complete set of DNA sequences of an organism and harbors all of the information required for that organism to function, including embryogenesis, growth, and responses to environmental cues. Unraveling such information means opening up its potential for human exploitation and uses. Although various techniques are now available to sequence a whole plant genome, the assembly of such information is difficult with most of the next-generation sequencing platforms either involving short read or long-read sequences (Eid et al., 2009; Clarke et al., 2009; Levy and Myers, 2016). High-throughput Chromosome Conformation Capture (Hi-C) is a chromosome conformation capture technique that can generate the input materials for deep sequencing and helps in assembling those associated DNA fragments and thus completely connecting chromosome structure and the genomic sequence (Belton et al., 2012). In this Research Topic, Zhou et al. reported a high-quality chromosome-scale genome assembly of *Quercus gilva* (a broad-leaf oak tree). This is the first reference genome for section Cyclobalanopsis, using the combination of Illumina and PacBio sequencing with Hi-C technologies. The assembled genome size of *Q. gilva* was 889.71 Mb with 36,442 protein-coding genes distributed on 12 pseudochromosomes. The analysis revealed that *Q. gilva* underwent considerable gene family expansion and contraction. *Ilex latifolia* Thunb. is a subtropical evergreen tree native to China and Japan. In addition to its ornamental functions, the tender leaves of this plant are processed into a specific kind of tea known as Kudingcha (Sun et al., 2011). The *Ilex latifolia* genome information (Xu et al.) along with transcriptome data predicted a total of 35,218 genes and three candidate genes for biosynthesis of pentacyclic triterpenoid saponins responsible for lowering blood lipid, lowering blood pressure, detoxification, and anti-cancer effects. *Amomum tsao-ko* (presently known as *Lanxangia tsaoko*) is an herbaceous plant of the Zingiberaceae family rich in volatile oils and is used in traditional Chinese medicine. Sun et al. prepared a draft genome (2.70 Gb in size, contig N50 of 2.45 Mb) of this medicinal plant using both of PacBio long reads and Illumina paired-end short reads and revealed significant expansion of genes involved in secondary metabolite biosynthesis. Notably, the 1-Deoxy-D-xylulose-5-phosphate synthase (*DXS*), geranylgeranyl diphosphate synthase (*GGPPS*), and cytochrome P450 (*CYP450*) genes were found to play a major role in essential oil formation in *amomum tsao-ko*.

## Research on fruit crops

Most of the fruit crops are highly cross-pollinating and with heterozygous genome makeup. Grafting is a well-developed vegetative propagation technique used for fruit trees in which a scion and rootstock are combined to form a new plant with a blend of each plant’s characteristics and is the most important reproduction mode for citrus production (Wu et al., 2019). In between rootstock and scion, horticulturists also use ‘interstock’ to control scion vigor, manage high-density orchard planting, and improve other important agronomic traits. Interstock grafting uses different genetic material between the rootstock and selected commercial cultivar, with the resulting plant formed from three different individuals through a double-graft union (Calderón et al., 2021). Liao et al. found interstocks help to regulate the early ripening and quality of citrus fruits by upregulating sucrose, fructose, and glucose contents, as well as by decreasing organic acid contents. Through transcriptome studies, they proved that the phytohormone signal is activated by alterations in the expression levels of *ERF1* (ethylene-responsive transcription factor 1B), *GA20OX2* (Gibberellic acid 20 oxidase), *CKI1* (CYTOKININ-INDEPENDENT1), and *TIR1* (TRANSPORT INHIBITOR RESPONSE 1). Genes related to sugar metabolism (i.e., starch, glucose, sucrose, fructose, and TCA cycle's metabolites) and energy metabolism or those encoding transcription factors (TFs) (e.g., MYB52, 547, and GRF5) were strongly affected by interstocks during fruit ripening. Another fruit plant papaya (*Carica papaya* L.) strengthens its heterozygosity by following the dioecy mechanism of pollination control. The sex in wild papaya is controlled by XY chromosomes, XX for females, and XY for males (Ming et al., 2008). Previous studies have suggested that sex differentiation in papaya may be regulated by transcription, epigenetic DNA methylation, and phytohormone by considering the experiments in above-ground parts (Zhou et al., 2020; Zhou P. et al., 2022). Zhou et al. in this Research Topic have used transcriptomics and metagenomics to reveal differential metabolites and soil microflora (bacteria and fungi) in the roots and rhizosphere of male and female papaya plants.

## Research on vegetable crops

Pakchoi (*Brassica rapa* subsp. *chinensis*) is an edible leafy vegetable, cultivated for its nutritional value, particularly with regard to vitamins, minerals, and dietary fibers (Jeon et al., 2018). Wu et al. observed the transcriptional and genomic structural alterations between diploid *B. rapa* (AA) and artificial autotetraploid *B. rapa* (AAAA) using RNA-seq and Hi-C techniques. In the autotetraploid *B. rapa*, eight differentially expressed genes (DEGs) with genomic structural variants were selected as potential candidate genes, including four DEGs involved in photosynthesis, three DEGs related to the chloroplast, and one DEG associated with disease resistance, which all showed high expression in this autotetraploid. An et al. used transcriptome sequencing in *Abelmoschus esculentus* to detect fruit color-related DEGs and reveal the biological processes and metabolic pathways associated with the related genes. Such detection of DEGs also facilitates the development of Expressed Sequenced Tags-Simple Sequence Repeat (EST-SSR) primer pairs to study genetic diversity in 153 A*. esculentus* varieties/lines and marker-trait association for fruit color. In other leafy vegetable crops, Li et al. established the role of two structural genes (dihydroflavonol-4-reductase/*DFR* and anthocyanidin synthase/*ANS*), three Glutathione S-Transferases (homologous to *TT19*), and 68 differentially expressed TFs, especially MYB-related TFs and WRKY44 in providing purple leaf color (rich in anthocyanins) in three *Brassica napus* varieties using metabolome and transcriptome approaches.

## Research on spice/medicinal crops

*Zanthoxylum bungeanum* Maxim. (genus: Zanthoxylum; family Rutaceae) has recently gained significant attention from researchers because of its applications in the pharmaceutical, food, and cosmetic industries (Sun et al., 2019). The spicy taste and medicinal properties of *Zanthoxylum bungeanum* are imparted by several alkylamides. Zhang et al. functionally validated the role of *ZmFAD2* and *ZmFAD3* in alkylamides production through their stable and transient expression in *Arabidopsis thaliana* and *Nicotiana benthamiana*. *Polygonatum cyrtonema* Hua is one of the most useful herbs in traditional Chinese medicine and a widely used medicinal and edible perennial plant. However, the seeds have the characteristics of epicotyl dormancy. Zhang et al. have established the role of higher content of trans-zeatin, proline, auxin, and gibberellin and lower content of flavonoids and arginine in relieving seed dormancy in 6-benzylaminopurine treated seeds through metabolomic and transcriptome study.

## Research on ornamentals

Ornamental crops have been a part of human life since civilization began due to their aesthetic appearance that indirectly help relieve human stress and improving the quality of space. *Chrysanthemum indicum* var. *aromaticum* has an intense fragrance, making it a novel resource plant for agricultural, medicinal, and industrial applications. Zhu et al. used integrative eal metabolome and transcriptome analyses at three different developmental stages of this special *Chrysanthemum* genotype to investigate key floral scent-related volatile compounds and genes in its flowers. Transcriptome analysis revealed significant DEGs and TFs involved in the production of volatile terpenes. *Bougainvillea* is known for its specialized, large, and colorful bracts, which contrast with its tiny colorless flower. Huang et al. employed a pan-transcriptome of bracts obtained from 18 *Bougainvillea glabra* accessions to investigate the global population level germplasm kinship and the gene regulation network for bract color variation. Transcriptome analysis revealed seven DEGs as an identifier of core regulation factors contributing to the *B. glabra* bract color variation. Orchidaceae family in ornamental crops harbor many premium orchids which are divided into epiphytic, terrestrial, and saprophytic types according to their life forms. Cellulose synthase (CesA) and cellulose synthase-like (Csl) genes are key regulators in the synthesis of plant cell wall polysaccharides, which play an important role in the adaptation of orchids to resist abiotic stresses, such as drought and cold. Wang et al. exploited available genome information from nine orchid species with three types of life forms (epiphytic, terrestrial, and saprophytic types) and detected eight subfamilies of cellulose synthase A/cellulose synthase-like (*CesA*/*Csl*) genes. Expansion of the CesA/Csl gene family in orchids mainly occurred in the CslD and CslF subfamilies. Of the three types of orchids, epiphytic orchids experienced greater strength of positive selection, with expansion events mostly related to the CslD subfamily, which might have resulted in strong adaptability to abiotic stress in epiphytes. *Dendrobium officinale* Kimura et Migo is a famous Chinese herb. *D. officinale* grows on rocks where the available phosphorus is low. The *SPX* family (*SYG1*, *PHO81*, and *Xpr1*) plays a critical role in maintaining Pi homeostasis in plants (Li et al., 2021). Liu et al. identified nine SPX family genes in the genome of *D. officinale* and studied their role in improving phosphorus content in stem through molecular interaction with the Phosphate High-Affinity Response factor in *Dendrobium* (DoPHR2). Availability of genome sequence in *Dendrobium officinale* assisted Wang et al. to identify 37 heat shock protein 20 (Hsp20) genes (*DenHsp20*s), 43 Hsp70 genes (*DenHsp70*s), and 4 Hsp90 genes (*DenHsp90*s). These genes were classified into 8, 4, and 2 subfamilies based on phylogenetic analysis and subcellular localization, respectively. Evolution genetics analysis revealed seven pairs in *DenHsp70*s were under positive selection and proved to be imparted strong stress tolerance in presence of methyl jasmonate. On the other hand, Jiao et al. revealed that treatment of protocorm-like bodies with terpenoid indole alkaloids precursors and methyl jasmonate significantly increased alkaloid production in *Dendrobium officinale*. Different time points transcriptome under the above treatment revealed six and seven genes related to alkaloid and JA biosynthetic pathways, respectively that might encode the key enzymes involved in the alkaloid biosynthesis of *D. officinale*. Moreover, 13 TFs, which mostly belong to AP2/ERF, WRKY, and MYB gene families, were predicted to regulate alkaloid biosynthesis.

## Research on industrial horticultural crops

Horticultural crops include a vast range of plants that are also used for industrial purposes. *The* Tung tree (*Vernicia fordii*), a unique industrial oil tree species in China, is a monoecious plant with wide distribution and many varieties (Cao et al., 2019). Jiang et al. studied the properties of young and old duplicate genes in *V. fordii* for the first time and identified important duplicate genes for imparting resistance to wilt disease. Jiang et al. analyzed five Euphorbiaceae species (generate raw materials for the production of biodiesel and rubber) genomes and evidenced novel mechanisms of controlling flowering in this species that does not contain *FRI* (*FRIGIDA*) and *FLC* (*FLOWERING LOCUS C*) genes. Wang et al. developed barcodes of different grass species of Gramineae by combining different barcoding genes that had a significantly higher identification effect than using a single fragment. These results met the requirements of DNA barcoding to locate species in a taxonomic system (family, genus, etc.) with sufficient phylogenetic information.

## Research on model crop species

Understanding of crop biology is often fueled by research on model crop plants. Model plant genomes have helped to isolate homologous genes in horticultural crops. In this Research Topic, we have displayed 12 research articles on model plants and/or established crop plants. Jun et al. summarize a new role of ARABIDOPSIS ELONGATOR PROTEIN 4 (*AtELP4*) in maintaining adaxial-abaxial polarity and cell proliferation during leaf development by epistatically act on DEFORMED ROOTS AND LEAVES 1 (*DRL1*). Before the birth of the genomics platform, we hardly know about the long noncoding RNAs (lncRNAs) that play an important regulatory role in the plant response to environmental stress. The soil in high-rainfall areas often becomes acidic due to the abundance of soluble aluminum. Thus, the aluminum tolerance mechanism is a required field of research in horticultural crops that are grown in such soils. The article of Gui et al. explains the role of two Al-activated-malate-transporter-related lncRNAs in *Medicago* in providing tolerance to aluminum toxicity by using a heterologous overexpressing yeast model. Similarly, Li et al. identified novel microRNAs in *Medicago sativa* that regulate phosphate starvation response by modulating target genes involved in carbohydrate metabolism, sulfo-lipid metabolism, glutathione metabolism, and hormone signal transduction. Saline-alkali soils pose an increasingly serious global threat to plant growth and productivity. Xiong et al. studied plant growth, transcriptional and metabolic responses of shoots to long-term potassium deficiency in maize and found that putrescine and putrescine derivatives were specifically accumulated in shoots under K deficiency. Besides, genes involved in K^+^ acquisition and homeostasis along with many stress-induced genes involved in transport, primary and secondary metabolism, and regulation were upregulated in maze shoots under K-deficiency. Ma et al. presented a review article on the molecular mechanism of plant responses to salt stress. They suggested deep research in establishing any connection between nutrient signaling and salt stress signaling to balance plant root growth and stress tolerance in plants.

Additionally, the role of growth-regulating factors (GRFs) in regulating leaf size was established in alfalfa (Sun et al.). On the other hand, Zheng et al. studied the architecture of TIFY family genes in casava and found that *MeJAZ1*, *MeJAZ13*, and *MeJAZ14* were highly up-regulated by osmotic, salt and cadmium treatments. Zhang et al. identified five upregulated WRKY genes, *AdWRKY18*, *AdWRKY40*, *AdWRKY42*, *AdWRKY56*, and *AdWRKY64* in *Arachis duranensis* under drought stress. Shi et al. studied the role of *Fusarium* toxin deoxynivalenol (DON) stress in the demethylation of the potato genome and subsequent transcript upregulation. They found that the differentially methylated region-associated DEGs were significantly enriched in resistance-related metabolic pathways and implicated the role of lower concentration of DON (5 and 35 ng/ml) in enhancing potato dry rot resistance through an unknown mechanism similar to seed priming. Dormancy is an important physiological attribute that controls the growth of propagules of many horticultural crops. Zhao et al. summarized the role of abscisic acid (ABA), gibberellic acid (GA), and light signaling in seed germination through the direct and indirect regulation of a core transcription factor ABSCISIC ACID INSENSITIVE 5 (ABI5) that represses seed germination in ABA signaling. The authors envisioned that future works should address the regulation of DELLA proteins by ABA, the crosstalk of ABA and GA in regulating ABI5, and regulations of ABI5 by PIF (phytochrome-interacting factors) and DELLA proteins. Li et al. used dynamic comparative transcriptomic analysis combined with weighted gene co-expression network analysis (WGCNA) to reveal key modules and hub genes related to the hardness and starch synthesis between the floury endosperm and the vitreous endosperm of wax corn structure and nutrient formation for the floury endosperm of maize. Zhang et al. summarized different cutting-edge technologies and theories including genome-wide association and genomic prediction using data collected from genomics and agronomic traits of agricultural crops and further discussed their utilities in horticultural crop breeding. Output from such studies will provide the key information and knowledge towards the input of the genome editing technology such CRISPR-Cas9 in many horticultural crops.

With increasing global climate change and a huge increase in the human population, there are severe problems and discrepancies between the global resources and the need of the human population, especially in places where the local population size is extremely large. People are facing these challenges and trying to solve the problems by improving the efficiency of agricultural production and keeping the balance between environmental capability and natural resources. To meet the need of the food requirements of the global population, diversification of crop husbandry is of prime concern. Cutting-edge genomics technologies, such as CRISPR-Cas9, have facilitated towards breeding of better breeds or varieties in major food crops (Menz et al., 2020; Pixley et al., 2022). It is now time to harvest the same magnitude of benefits in horticultural crops.

## Author contributions

All authors have made a substantial, direct, and intellectual contribution toward shaping this editorial and approved it for communication.

## Acknowledgments

Editors thank all the contributing authors in this Research Topic.

## Conflict of interest

The authors declare that the research was conducted in the absence of any commercial or financial relationships that could be construed as a potential conflict of interest.

## Publisher’s note

All claims expressed in this article are solely those of the authors and do not necessarily represent those of their affiliated organizations, or those of the publisher, the editors and the reviewers. Any product that may be evaluated in this article, or claim that may be made by its manufacturer, is not guaranteed or endorsed by the publisher.
